# Dynamics of Anti-HBc Serology During Anti-CD20 Therapy: Incidence, Timing, and Predictors of Anti-HBc Seroclearance in a Retrospective Cohort

**DOI:** 10.3390/medicina62091659

**Published:** 2026-08-29

**Authors:** Fatih Eren, Muhammed Abdurrahman Celik, Resul Akduman, Mete Yasar, Mehmet Refik Goktug, Gokhan Ocakoglu, Selim Gurel

**Affiliations:** 1 Division of Gastroenterology, Department of Internal Medicine, Faculty of Medicine, Bursa Uludag University, Bursa 16059, Turkey; drmuhammedcellik@gmail.com (M.A.C.); resulakduman@uludag.edu.tr (R.A.); meteyasar@uludag.edu.tr (M.Y.); mrgoktug@hotmail.com (M.R.G.); gurels@uludag.edu.tr (S.G.); 2 Department of Biostatistics, Faculty of Medicine, Bursa Uludag University, Bursa 16059, Turkey; ocakoglu@uludag.edu.tr

**Keywords:** hepatitis B virus, anti-HBc seroclearance, anti-CD20 therapy, HBsAg-negative/anti-HBc-positive, HBV reactivation, mycophenolate mofetil

## Abstract

*Background and Objectives:* Anti-hepatitis B core antibody (anti-HBc) is generally regarded as a lifelong serological marker of previous hepatitis B virus (HBV) infection. However, anti-HBc seroclearance has been increasingly reported in patients receiving anti-CD20 therapy, while its incidence, longitudinal serological dynamics, and clinical significance remain insufficiently characterized. In this study, we aimed to evaluate the incidence, timing, and predictors of anti-HBc seroclearance and investigate longitudinal changes in HBV serology in HBsAg-negative/anti-HBc-positive patients undergoing anti-CD20 therapy. *Materials and Methods:* This retrospective single-center cohort study included 230 HBsAg-negative/anti-HBc-positive adults who received anti-CD20 therapy between 2011 and 2025. Anti-HBc seroclearance was evaluated using Kaplan–Meier analysis and Cox proportional hazards regression. Longitudinal changes in anti-HBc and anti-HBs titers were assessed using linear mixed-effects models. *Results:* Confirmed anti-HBc seroclearance occurred in 38 of 230 patients (16.5%). Younger age (adjusted hazard ratio (aHR) 0.96, 95% confidence interval (CI) 0.93–0.98; *p* = 0.001), fewer anti-CD20 treatment cycles (aHR 0.90, 95% CI 0.83–0.99; *p* = 0.025), and mycophenolate mofetil therapy (aHR 2.52, 95% CI 1.14–5.56; *p* = 0.022) were independently associated with anti-HBc seroclearance. Anti-HBc titers progressively declined during follow-up, with a significantly steeper decline among patients receiving mycophenolate mofetil. HBV reactivation occurred in five of 230 patients (2.2%), including two who had developed anti-HBc seroclearance. HBsAg seroreversion occurred in all five patients. *Conclusions:* Anti-HBc seroclearance is a relatively frequent serological finding in HBsAg-negative/anti-HBc-positive patients receiving anti-CD20 therapy and is accompanied by progressive declines in anti-HBc titers. Despite seroclearance, HBV reactivation may still occur, indicating that anti-HBc loss should not be considered evidence of elimination of HBV-related immunological risk. These findings improve our understanding of HBV serological dynamics during anti-CD20 therapy and support maintaining current antiviral prophylaxis and virological monitoring strategies regardless of anti-HBc seroclearance.

## 1. Introduction

Hepatitis B virus (HBV) infection is not completely eradicated after HBsAg clearance, as covalently closed circular DNA (cccDNA) may persist within hepatocytes for years [1,2]. The serological profile after HBsAg loss is heterogeneous; anti-HBc generally indicates previous or current HBV infection, while anti-HBs may or may not be detectable. Moreover, occult HBV infection, defined by the presence of replication-competent HBV DNA in the liver and, in some cases, detectable HBV DNA in serum despite HBsAg negativity, may occur with or without detectable anti-HBc and/or anti-HBs. Therefore, HBsAg-negative individuals with evidence of previous HBV exposure may remain at risk of HBV reactivation during profound immunosuppression [3].

Rituximab and other anti-CD20 monoclonal antibodies that target B lymphocytes are widely used in the treatment of hematologic malignancies, as well as numerous rheumatologic, neurologic, nephrologic, and dermatologic disorders. Owing to their prolonged B-cell-depleting effects, these agents are considered among the immunosuppressive therapies associated with the highest risk of HBV reactivation [4,5]. Prospective and retrospective cohort studies have reported clinically significant HBV reactivation and HBsAg seroreversion following rituximab therapy in HBsAg-negative/anti-HBc-positive patients who did not receive antiviral prophylaxis [6,7]. Based on this robust body of evidence, current international guidelines recommend antiviral prophylaxis for HBsAg-negative/anti-HBc-positive patients undergoing anti-CD20 therapy [3,8].

Anti-HBc is a serological marker of previous or current HBV infection and has traditionally been assumed to remain detectable throughout life. However, recent studies in immunocompromised patients have demonstrated that anti-HBc antibodies may disappear either permanently or transiently [9]. In particular, reports of anti-HBc seroclearance in patients receiving anti-CD20 therapy suggest that the serological profile of previous HBV infection may be more dynamic than previously recognized [10].

Nevertheless, the available evidence remains limited and heterogeneous, and confirmed anti-HBc seroclearance has rarely been evaluated as a predefined primary endpoint in anti-CD20-treated cohorts [9,10]. The true incidence, timing, clinical course, and associated risk factors of anti-HBc seroclearance during rituximab therapy remain inadequately characterized. Furthermore, the potential clinical implications of anti-HBc seroclearance for antiviral prophylaxis, HBV serological surveillance, and future immunosuppressive treatment decisions remain uncertain.

Moreover, anti-HBc seroclearance may represent only the final stage of changes in HBV-specific humoral immunity. Even in patients who do not develop seroclearance, anti-HBc levels may progressively decline over time, potentially reflecting gradual impairment of HBV-specific humoral immunity induced by anti-CD20 therapy. Therefore, serial assessment of anti-HBc may provide a more comprehensive understanding of the temporal dynamics of anti-HBc serology. Although anti-HBs provides complementary information regarding HBV-specific humoral immunity, the primary focus of the present study was anti-HBc because the loss of this traditionally persistent marker during anti-CD20 therapy remains poorly characterized. Anti-HBs dynamics were therefore evaluated as a complementary secondary outcome.

This study aimed to evaluate the incidence, timing, and clinical determinants of anti-HBc seroclearance in HBsAg-negative/anti-HBc-positive patients receiving anti-CD20 therapy. We also sought to characterize the longitudinal changes in anti-HBc serology and investigate the association between these serological changes and HBV reactivation.

## 2. Materials and Methods

### 2.1. Study Design and Patient Selection

This retrospective, single-center cohort study was conducted at Bursa Uludağ University Faculty of Medicine, Bursa, Turkey. Adult patients who received rituximab or another anti-CD20 monoclonal antibody between 7 March 2011 and 30 September 2025 were retrospectively identified through the institutional electronic medical record system. Clinical characteristics, laboratory findings, treatment-related variables, and HBV serological follow-up data were collected through 26 June 2026, which served as the administrative censoring date.

Patients aged ≥18 years were eligible if they had an HBsAg-negative/anti-HBc-positive serological profile before initiation of anti-CD20 therapy and had sufficient post-treatment follow-up to permit longitudinal evaluation of anti-HBc serology. Anti-CD20 therapy was administered for various clinical indications, including hematologic, rheumatologic, neurologic, nephrologic, and dermatologic diseases.

**Figure 1:**
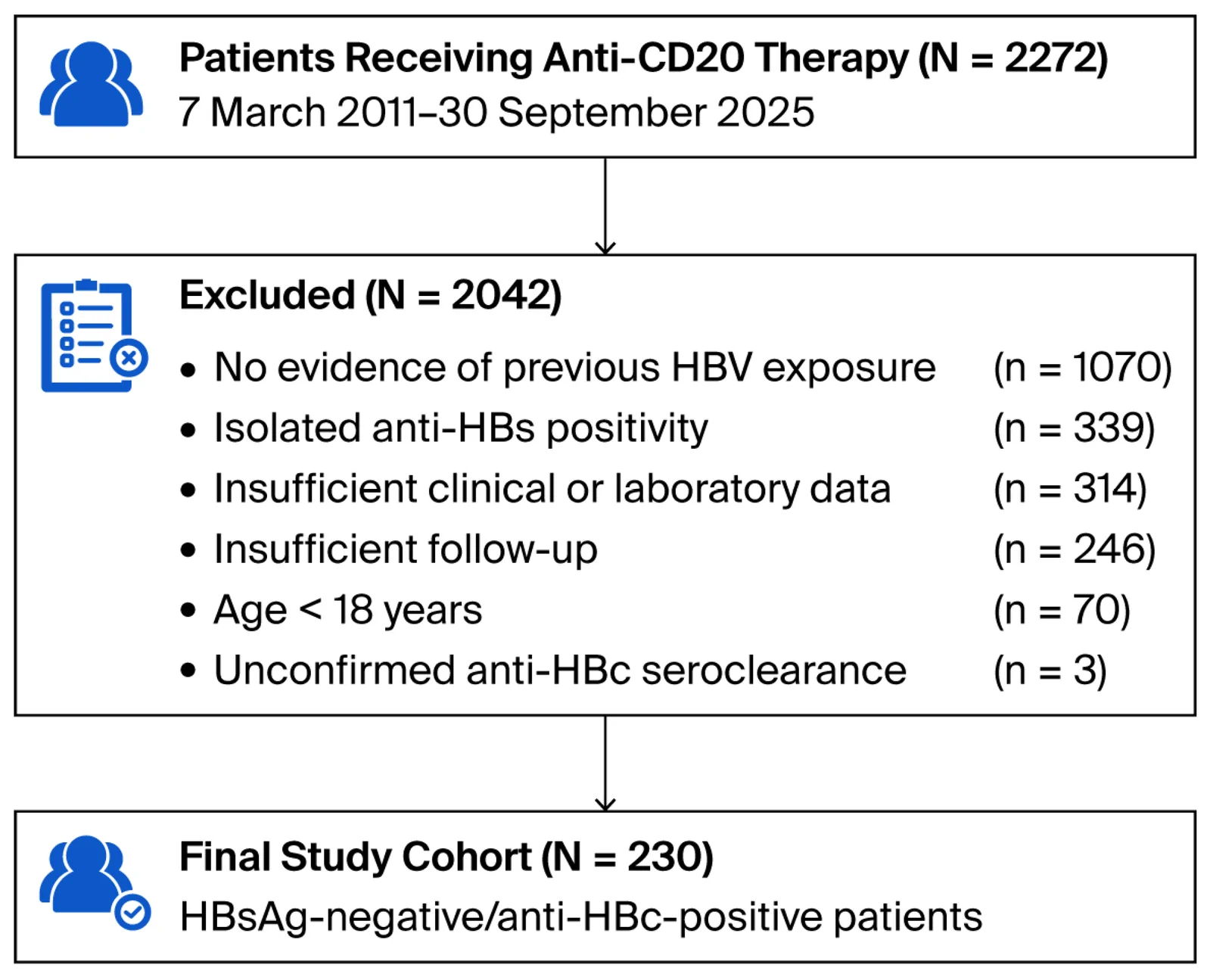
Flow diagram of patient selection.

The exclusion criteria include an age of <18 years, absence of an HBsAg-negative/anti-HBc-positive serological profile, isolated anti-HBs positivity suggestive of vaccination alone, insufficient post-treatment follow-up for the evaluation of anti-HBc serology, or incomplete clinical or laboratory data. After application of the eligibility criteria, 230 patients were included in the final study cohort. The patient selection process is illustrated in Figure 1.

### 2.2. Data Collection, Follow-Up, and Treatment Exposure

Demographic, clinical, treatment-related and laboratory data were retrospectively extracted from the hospital’s electronic medical record system. Demographic variables included age and sex. Clinical variables included the indication for anti-CD20 therapy, underlying disease, comorbidities, history of hematopoietic stem cell transplantation, and use of intravenous immunoglobulin.

Treatment-related variables included the anti-CD20 agent administered, the date of treatment initiation, the date of the last treatment cycle, the total number of treatment cycles, and concomitant immunosuppressive or immunomodulatory therapies. Information regarding antiviral prophylaxis included whether prophylaxis was initiated, the antiviral agent used, and any modification of antiviral therapy during follow-up. Antiviral agents were categorized as entecavir, tenofovir disoproxil fumarate, tenofovir alafenamide, or lamivudine. For patients who received more than one antiviral agent during follow-up, each antiviral exposure was recorded separately.

Laboratory assessments included alanine aminotransferase, aspartate aminotransferase, total bilirubin, albumin, creatinine, and complete blood count parameters, including leukocyte count, absolute neutrophil count, absolute lymphocyte count, hemoglobin concentration, and platelet count. HBV-related serological and virological evaluations included HBsAg, anti-HBs, anti-HBc, and HBV DNA results. HBV serological markers were predominantly analyzed using chemiluminescent microparticle immunoassays on the Alinity i immunoassay system (Abbott Diagnostics, Abbott Park, Chicago, IL, USA). A small proportion of earlier baseline measurements were performed using the immunoassay platform in routine use at the institution at that time. Importantly, all confirmatory anti-HBc measurements used to verify seroclearance were performed using the Alinity i system. Results were interpreted according to the manufacturer-specific assay cutoffs. Serum HBV DNA was quantified using a real-time polymerase chain reaction assay on the Alinity m system (Abbott Molecular, Des Plaines, IL, USA), with a quantification range of 10 to 10^9^ IU/mL (1.0–9.0 log10 IU/mL).

HBV serological follow-up was analyzed relative to the initiation of anti-CD20 therapy. Anti-HBc and anti-HBs were assessed at baseline and subsequently at approximately 6-month intervals. When available, measurements obtained at 6, 12, 18, 24, 30, 36, 42, 48, and 60 months were recorded for longitudinal assessment. Because of the inherent variability in real-world clinical practice, serological measurements were not available for every patient at every scheduled time point.

Anti-CD20 therapy was administered by the respective specialty departments according to contemporary national and international guidelines and standard clinical practice, based on each patient’s underlying disease. Treatment regimens varied according to the underlying condition and therapeutic indication. In patients with hematologic diseases, the most commonly used rituximab regimens were four weekly doses of 375 mg/m^2^ or two 1000 mg doses administered 2 weeks apart. In patients with rheumatologic and other immune-mediated diseases, the standard regimen generally consisted of two 1000 mg doses given 2 weeks apart. When clinically indicated, retreatment or maintenance courses were administered according to disease activity and treatment response.

To minimize the heterogeneity arising from differences in dosing schedules and administration protocols across indications, treatment exposure was quantified using the total number of anti-CD20 treatment cycles rather than the cumulative rituximab dose. In patients treated with anti-CD20 monoclonal antibodies other than rituximab, exposure to all anti-CD20 agents was recorded, and treatment cycles were collectively analyzed as total anti-CD20 exposure.

Antiviral prophylaxis was administered in accordance with the prevailing national and international HBV guidelines applicable during the treatment period, using standard adult doses of entecavir (0.5 mg/day), tenofovir disoproxil fumarate (300 mg/day), tenofovir alafenamide (25 mg/day), or lamivudine (100 mg/day), with dose or dosing interval adjustments in patients with impaired renal function as clinically indicated.

### 2.3. Definitions

The baseline HBV serological profile was defined by HBsAg negativity and anti-HBc positivity before initiation of anti-CD20 therapy. Baseline anti-HBs positivity was not required for study eligibility. Baseline serum HBV DNA was assessed in all patients before initiation of anti-CD20 therapy.

Anti-HBc seroclearance was defined as the loss of previously detectable anti-HBc during follow-up, with the anti-HBc result falling below the predefined assay cutoff (S/CO < 1.0). The date of seroclearance was defined as the date of the first negative anti-HBc result. Seroclearance was confirmed by a subsequent serological assessment. Anti-HBc seroclearance was classified as occurring during active anti-CD20 treatment when the first documented anti-HBc-negative result occurred on or before the recorded treatment end date, and as post-treatment when it occurred after the treatment end date.

To account for the potential passive transfer of anti-HBc following intravenous immunoglobulin (IVIG) administration, the temporal relationship between IVIG exposure and the first documented anti-HBc-positive result was individually reviewed in all patients who received IVIG. Patients with documented anti-HBc positivity before IVIG exposure were considered to have pre-existing anti-HBc positivity. Given that the probability of passively transferred anti-HBc positivity has been shown to decline substantially by approximately 12 weeks after IVIG administration [11], a sensitivity analysis was performed after excluding patients whose first documented anti-HBc-positive result occurred within 90 days after IVIG administration.

HBV reactivation was defined as the reappearance of detectable serum HBV DNA during or after anti-CD20 therapy and/or re-emergence of HBsAg in patients who were HBsAg-negative at baseline (HBsAg seroreversion). HBV reactivation was assessed in accordance with current international guideline recommendations.

HBsAg seroreversion was defined as the reappearance of HBsAg during follow-up in patients who were HBsAg-negative at baseline.

Anti-HBs loss was defined as the loss of detectable anti-HBs during follow-up in patients who were anti-HBs-positive at baseline.

### 2.4. Study Outcomes

The primary endpoints of the study were the incidence of anti-HBc seroclearance following anti-CD20 therapy and the time to the development of anti-HBc seroclearance.

Secondary endpoints included HBV reactivation, HBsAg seroreversion, anti-HBs loss, longitudinal changes in anti-HBc and anti-HBs titers, characteristics related to antiviral prophylaxis, and the clinical and laboratory factors associated with anti-HBc seroclearance. In addition, the effects of age, sex, underlying disease category, antiviral agent, number of anti-CD20 treatment cycles, history of hematopoietic stem cell transplantation, intravenous immunoglobulin use, and absolute lymphocyte count on the development of anti-HBc seroclearance were evaluated. Longitudinal trends in anti-HBc and anti-HBs titers were assessed descriptively in patients with and without anti-HBc seroclearance.

### 2.5. Statistical Analysis

Statistical analyses were performed using IBM SPSS Statistics version 25.0 (IBM Corp., Armonk, Westchester County, NY, USA) and R version 4.5.0 (R Foundation for Statistical Computing, Vienna, Austria). Continuous variables were assessed for normality using the Shapiro–Wilk test. Normally distributed continuous variables are presented as mean ± standard deviation, whereas non-normally distributed variables are summarized as median (interquartile range). Categorical variables are expressed as counts and percentages. Group comparisons were performed using the Mann–Whitney U test for continuous variables and the chi-square test or Fisher’s exact test for categorical variables, as appropriate. Two-sided *p*-values below 0.05 were considered statistically significant.

Time-to-event analyses were performed using Kaplan–Meier methods, and differences between groups were evaluated using the log-rank test. Time to anti-HBc seroclearance was defined as the interval from anti-CD20 therapy initiation to the first documented anti-HBc-negative result (S/CO < 1.0). Participants without anti-HBc seroclearance were censored at the date of their last available follow-up. Predictors of anti-HBc seroclearance were evaluated using Cox proportional hazards regression. For patients who developed anti-HBc seroclearance, cumulative anti-CD20 exposure was calculated using only treatment cycles administered before the date of the first documented anti-HBc-negative result. For patients without seroclearance, cumulative exposure was calculated up to the last available follow-up date. Univariable hazard ratios were estimated using separate Cox proportional hazards regression models. Rheumatologic and neurologic disease categories were analyzed as categorical variables using patients without rheumatologic or neurologic disease as the respective reference groups. Mycophenolate mofetil exposure was defined as treatment initiated before or concomitantly with anti-CD20 therapy and continued during the treatment period. Variables demonstrating statistical significance in univariable analyses and variables considered clinically relevant were selected for multivariable Cox regression, while taking the number of outcome events and events-per-variable considerations into account. Proportional hazards assumptions were assessed using Schoenfeld residual testing. A sensitivity analysis was also performed after excluding patients with detectable baseline HBV DNA to assess whether their inclusion influenced the observed anti-HBc seroclearance rate.

Longitudinal changes in anti-HBc and anti-HBs titers were evaluated using linear mixed-effects models with patient-specific random intercepts to account for repeated within-subject measurements. Because the number of available observations progressively declined during extended follow-up, resulting in substantial attrition beyond month 24, the primary longitudinal mixed-effects analyses of anti-HBc and anti-HBs titers were restricted to the first 24 months after anti-CD20 therapy initiation to ensure adequate representation across study visits and improve the stability of model estimates. Serological measurements beyond 24 months were retained for descriptive purposes only and were not included in the mixed-effects models. Anti-HBc and anti-HBs titers were transformed as log10 (titer + 1) before modeling to accommodate zero or near-zero values. Fixed effects included follow-up time, mycophenolate mofetil (MMF) exposure, and the interaction between follow-up time and MMF exposure. Estimated marginal means were calculated to visualize model-derived trajectories over time.

## 3. Results

### 3.1. Baseline Characteristics

A total of 230 HBsAg-negative/anti-HBc-positive patients were included in the final study cohort. Baseline serum HBV DNA was available in all 230 patients; HBV DNA was undetectable in 220 patients (95.7%) and detectable in 10 patients (4.3%). None of the 10 patients with detectable baseline HBV DNA developed anti-HBc seroclearance. In a sensitivity analysis restricted to the 220 patients with undetectable baseline HBV DNA, confirmed anti-HBc seroclearance occurred in 38 patients (17.3%), compared with 16.5% in the overall cohort. During follow-up, confirmed anti-HBc seroclearance occurred in 38 patients (16.5%), whereas 192 patients (83.5%) remained persistently anti-HBc-positive. Three additional patients were initially classified as having anti-HBc seroclearance but were subsequently excluded because no laboratory record confirmed an anti-HBc level below the assay cutoff (S/CO < 1.0). All 38 confirmed anti-HBc seroclearance events were subsequently verified by at least one additional anti-HBc-negative measurement. Baseline demographic, clinical, treatment-related, and laboratory characteristics are summarized in Table 1.

Among the 38 patients who received IVIG, 22 had documented anti-HBc positivity before IVIG exposure. Four patients had their first documented anti-HBc-positive result within 90 days after IVIG administration; none of these patients subsequently developed anti-HBc seroclearance. In a sensitivity analysis excluding these four patients, the overall seroclearance rate remained essentially unchanged (38/226, 16.8%), compared with 38/230 (16.5%) in the primary cohort.

Regarding the timing of seroclearance in relation to anti-CD20 exposure, 31 of the 38 patients (81.6%) developed anti-HBc seroclearance during active anti-CD20 treatment, whereas 7 (18.4%) developed seroclearance after treatment completion.

### 3.2. Longitudinal Dynamics of Anti-HBc and Anti-HBs Serology

Longitudinal changes in anti-HBc and anti-HBs titers according to anti-HBc seroclearance status are summarized in Table 2. Patients who developed anti-HBc seroclearance demonstrated a progressive decline in anti-HBc titers throughout follow-up, whereas anti-HBc titers remained relatively stable in patients without seroclearance. In contrast, anti-HBs titers generally increased in the seroclearance group but tended to decline among patients without seroclearance. Because the number of available observations progressively decreased over time, later follow-up results should be interpreted with caution.

The results of the univariable and multivariable Cox proportional hazards regression analyses are summarized in Table 3. The final model included age, cumulative anti-CD20 exposure, history of hematopoietic stem cell transplantation, and mycophenolate mofetil therapy (Table 3).

In univariable analyses, younger age, fewer cumulative anti-CD20 treatment cycles, history of hematopoietic stem cell transplantation, mycophenolate mofetil therapy, IVIG therapy, and rheumatologic disease category were associated with anti-HBc seroclearance. Regarding nucleos(t)ide analog prophylaxis, tenofovir alafenamide exposure was associated with a higher rate of anti-HBc seroclearance in univariable analysis (HR 2.03, 95% CI 1.06–3.89; *p* = 0.033), whereas entecavir and tenofovir disoproxil fumarate exposure was not significantly associated with seroclearance. The effect of lamivudine exposure could not be reliably estimated because of the small number of exposed patients and the absence of seroclearance events in this subgroup. In the multivariable Cox proportional hazards model, younger age remained independently associated with anti-HBc seroclearance (HR 0.96, 95% CI 0.93–0.98, *p* = 0.001). A lower cumulative number of anti-CD20 treatment cycles also remained independently associated with seroclearance (HR 0.90, 95% CI 0.83–0.99, *p* = 0.025). Mycophenolate mofetil therapy was independently associated with an increased rate of anti-HBc seroclearance (HR 2.52, 95% CI 1.14–5.56, *p* = 0.022). History of hematopoietic stem cell transplantation showed a borderline association after adjustment (HR 2.89, 95% CI 0.97–8.64, *p* = 0.057). No other variables retained statistical significance after multivariable adjustment. No evidence of a violation of the proportional hazards assumption was observed for the final multivariable model (global Schoenfeld residual test, *p* = 0.380).

Because mycophenolate mofetil therapy and IVIG therapy demonstrated a strong association, only mycophenolate mofetil therapy was retained in the final multivariable model to minimize treatment-related collinearity.

**Figure 2:**
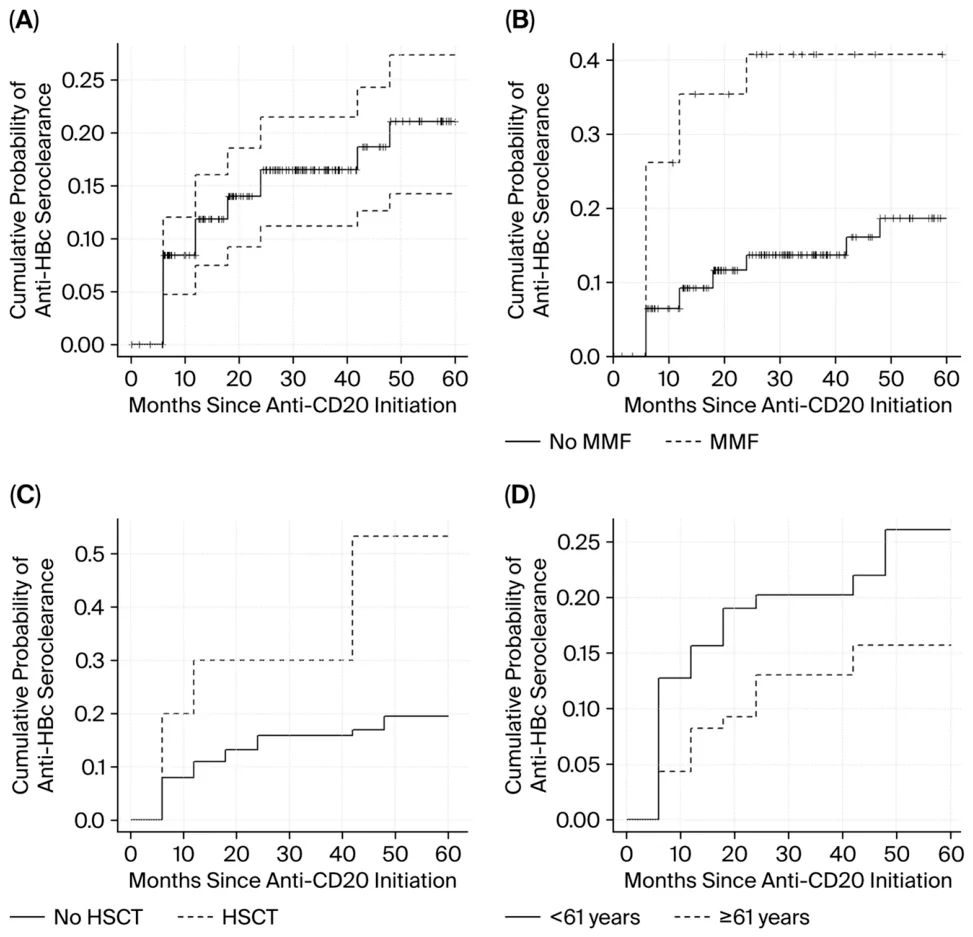
Kaplan–Meier estimates of the cumulative probability of anti-HBc seroclearance. (**A**) Overall cohort; (**B**) according to mycophenolate mofetil (MMF) exposure; (**C**) according to hematopoietic stem cell transplantation (HSCT) history; and (**D**) according to age group (<61 vs. ≥61 years). Groups in panels B–D were compared using the log-rank test. Tick marks indicate censored observations.

Figure 2 illustrates the cumulative probability of anti-HBc seroclearance following initiation of anti-CD20 therapy. Among 230 eligible participants, 38 confirmed anti-HBc seroclearance events were observed during follow-up. The cumulative probability of anti-HBc seroclearance increased progressively over time, reaching 8.4% at 6 months, 11.9% at 12 months, 14.0% at 18 months, 16.5% at 24 months, 18.7% at 42 months, and 21.1% at 48 months. No additional seroclearance events were observed thereafter. Throughout the study period, the cumulative probability of anti-HBc seroclearance remained below 50%; consequently, the median time to anti-HBc seroclearance was not reached.

### 3.3. Kaplan–Meier Analyses According to Clinical Predictors

Kaplan–Meier analyses were performed according to mycophenolate mofetil therapy, history of hematopoietic stem cell transplantation, and age group (Figure 2B–D). Patients receiving mycophenolate mofetil therapy demonstrated a significantly higher cumulative probability of anti-HBc seroclearance than those not receiving mycophenolate mofetil therapy (log-rank *p* < 0.001; Figure 2B). Anti-HBc seroclearance was observed in 9 of 23 patients (39.1%) receiving mycophenolate mofetil therapy, compared with 29 of 207 patients (14.0%) who did not receive it. Consistent with the Cox regression findings, separation of the Kaplan–Meier curves was observed early during follow-up and persisted throughout the observation period.

In addition, participants with a history of hematopoietic stem cell transplantation had a higher cumulative probability of anti-HBc seroclearance than patients without prior transplantation (log-rank *p* = 0.042; Figure 2C). Anti-HBc seroclearance occurred in 3 of 8 participants (37.5%) with a history of transplantation, compared with 35 of 222 participants (15.8%) without such a history.

When the cohort was stratified by the median age of 61 years, younger patients showed a numerically higher cumulative probability of seroclearance throughout follow-up; however, the between-group difference did not reach conventional statistical significance in the log-rank analysis (*p* = 0.075; Figure 2D). Anti-HBc seroclearance was documented in 24 of 114 patients (21.1%) younger than 61 years and in 14 of 116 patients (12.1%) aged 61 years or older.

Accordingly, the median time to anti-HBc seroclearance was not reached in any subgroup analysis.

### 3.4. Longitudinal Dynamics of Anti-HBc Titers

**Figure 3:**
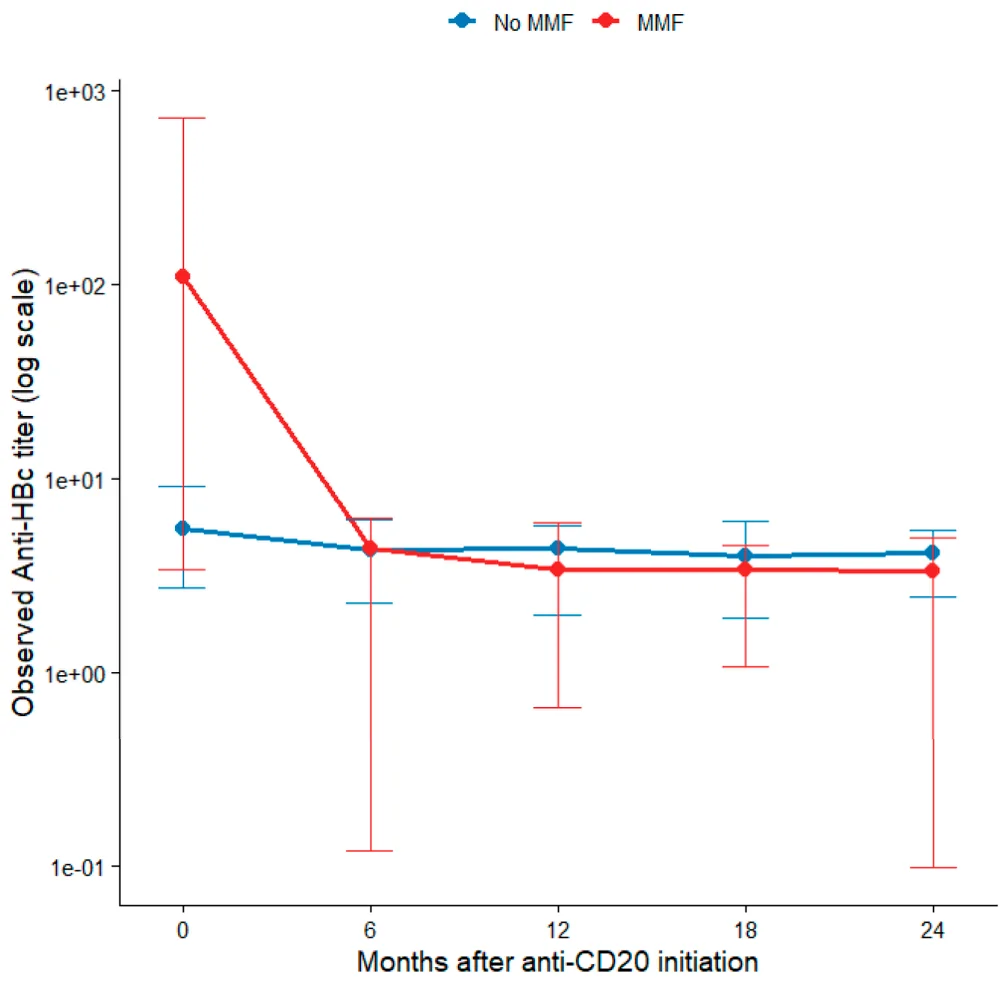
Observed anti-HBc titers by follow-up visit according to MMF exposure. The *y*-axis is displayed on a logarithmic scale because of substantial baseline variability and extreme anti-HBc titers.

**Figure 4:**
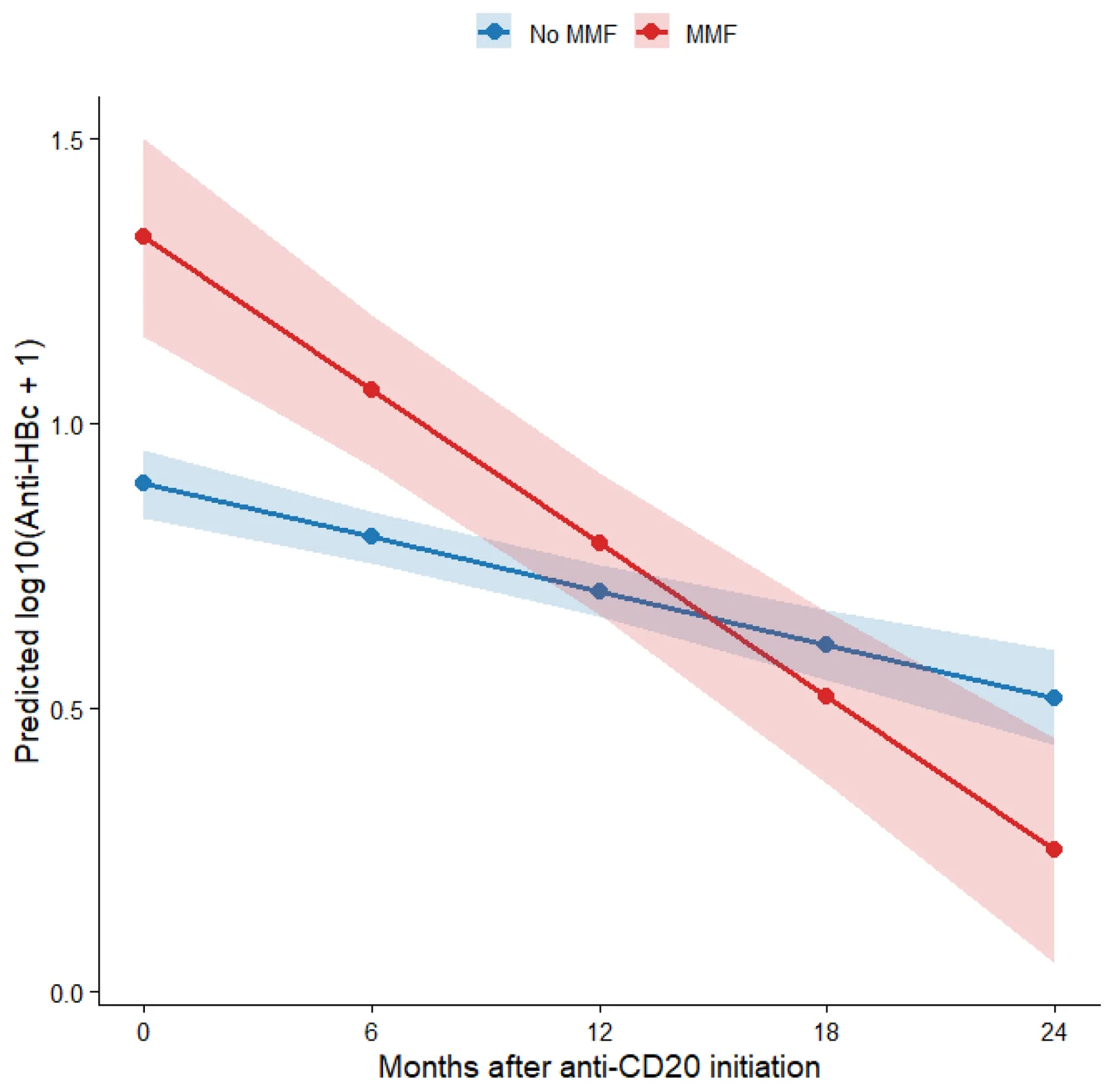
Model-derived longitudinal anti-HBc trajectories according to mycophenolate mofetil (MMF) exposure during the first 24 months following anti-CD20 therapy initiation. Shaded areas indicate 95% confidence intervals.

To further characterize the temporal evolution of anti-HBc titers following anti-CD20 therapy, a longitudinal analysis was performed using repeated anti-HBc measurements obtained during follow-up. Both observed anti-HBc trajectories (Figure 3) and model-derived longitudinal estimates (Figure 4) demonstrate a gradual decline in anti-HBc titers over time. The corresponding descriptive statistics and mixed-effects model results are presented in Table 4.

Anti-HBc titers decreased progressively during follow-up after initiation of anti-CD20 treatment. Median anti-HBc levels declined from 5.70 S/CO (IQR, 2.90–12.90) at baseline to 4.30 S/CO (IQR, 2.30–6.14) at month 6, 4.30 S/CO (IQR, 1.90–5.70) at month 12, 3.99 S/CO (IQR, 1.77–5.82) at month 18, and 3.85 S/CO (IQR, 2.02–5.38) at month 24 (Table 4; Figure 3).

In the linear mixed-effects model, follow-up time was significantly associated with declining anti-HBc titers (F = 88.36, *p* < 0.001). Mycophenolate mofetil use was independently associated with anti-HBc dynamics (F = 21.24, *p* < 0.001). A significant interaction between follow-up time and MMF exposure was also observed (F = 20.58, *p* < 0.001), indicating that the rate of anti-HBc decline differed according to MMF use. Model-derived predicted trajectories demonstrated a consistently steeper decline among patients receiving MMF compared with those not receiving MMF throughout the first 24 months of follow-up (Figure 4).

### 3.5. Longitudinal Dynamics of Anti-HBs Titers

**Figure 5:**
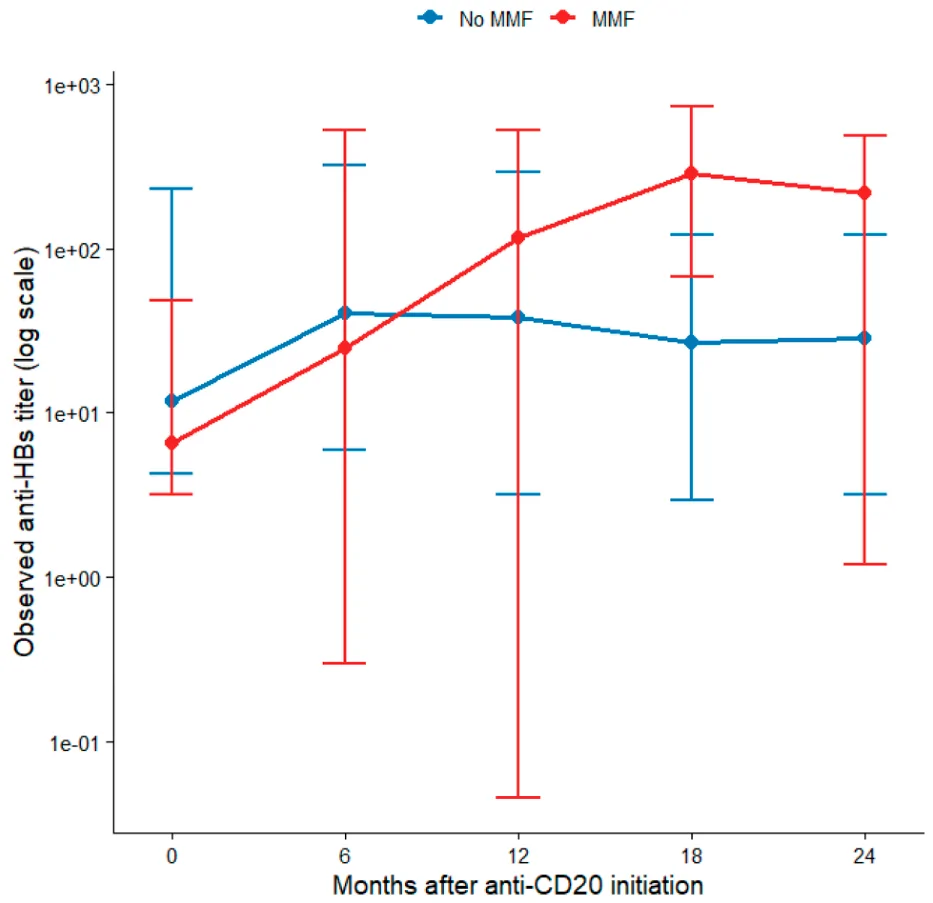
Observed anti-HBs titers by follow-up visit according to MMF exposure. Observed median anti-HBs titers and interquartile ranges are displayed for each follow-up visit. The *y*-axis is displayed on a logarithmic scale because of substantial inter-individual variability and the presence of extreme anti-HBs values.

**Figure 6:**
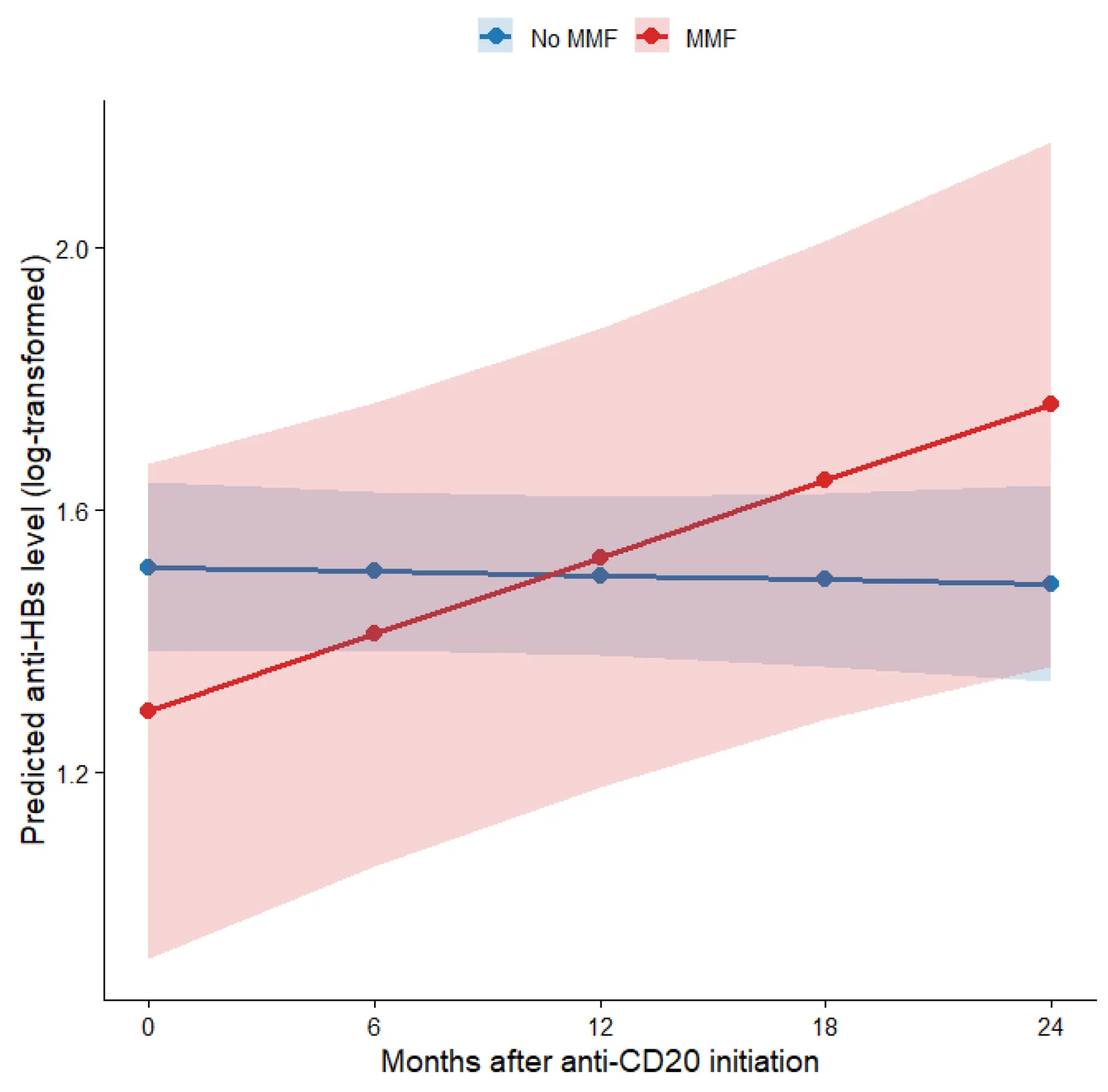
Model-derived longitudinal anti-HBs trajectories according to mycophenolate mofetil (MMF) exposure during the first 24 months after anti-CD20 therapy initiation. Symbols represent estimated marginal means and shaded areas indicate 95% confidence intervals.

To further characterize longitudinal changes in anti-HBs titers following anti-CD20 therapy, repeated anti-HBs measurements obtained during follow-up were evaluated. Both observed anti-HBs trajectories (Figure 5) and model-derived longitudinal estimates (Figure 6) were assessed to characterize temporal changes in anti-HBs titers. The corresponding descriptive statistics and mixed-effects model results are summarized in Table 5.

Anti-HBs titers changed over the course of follow-up. Median anti-HBs levels increased from 10.1 mIU/mL (IQR, 4.12–192.0) at baseline to 38.3 mIU/mL (IQR, 5.85–361.0) at month 6 and 39.1 mIU/mL (IQR, 2.35–300.0) at month 12, followed by relative stabilization through months 18 and 24 (Table 5; Figure 5).

In the linear mixed-effects model, follow-up time was significantly associated with longitudinal anti-HBs dynamics (F = 5.41, *p* = 0.020). MMF exposure was not independently associated with overall anti-HBs levels (F = 1.17, *p* = 0.280). However, a significant interaction between follow-up time and MMF exposure was observed (F = 6.74, *p* = 0.010), indicating that the temporal pattern of anti-HBs changes differed according to MMF use. Consistent with this finding, model-based predicted trajectories demonstrated distinct anti-HBs kinetics between MMF-exposed and non-exposed patients during follow-up (Figure 6).

### 3.6. HBV Reactivation and Clinical Outcomes

During follow-up, HBV reactivation occurred in 5 of 230 patients (2.2%). HBV reactivation occurred in 2 of 38 patients (5.3%) with anti-HBc seroclearance and in 3 of 192 patients (1.6%) with persistent anti-HBc positivity; this difference was not statistically significant (Fisher’s exact test, *p* = 0.192). HBsAg seroreversion was observed in all five patients, whereas HBV-related hepatitis developed in three patients (60%). HBV DNA levels at the time of reactivation varied markedly, ranging from undetectable to 709,628,151 IU/mL. Among the five patients with HBV reactivation, antiviral prophylaxis was ongoing at the time of reactivation in two patients, whereas it had been discontinued or interrupted in three patients. Among the three patients who developed HBV-related hepatitis, two had discontinued or interrupted antiviral prophylaxis before reactivation.

Anti-HBc seroclearance was documented in two of the five patients with HBV reactivation. Regarding clinical outcomes, two patients remained alive without liver-related complications during follow-up, whereas two patients died from liver-related causes (HBV-related hepatitis and acute-on-chronic liver failure). One additional patient died due to progression of the underlying hematologic malignancy. Clinical characteristics, management, and outcomes of patients with HBV reactivation are summarized in Table 6.

## 4. Discussion

Anti-HBc is widely recognized as one of the most reliable serological markers of previous HBV infection, and current international guidelines classify HBsAg-negative/anti-HBc-positive individuals as being at risk for HBV reactivation [3,5,8]. However, emerging evidence demonstrating that anti-HBc seroclearance can occur during anti-CD20 therapy challenges this traditional paradigm and suggests that the long-held assumption of lifelong anti-HBc persistence warrants reconsideration [9,10]. Importantly, HBV reactivation and clinically significant adverse outcomes were observed even among patients who developed anti-HBc seroclearance, indicating that the loss of detectable anti-HBc should not be interpreted as evidence of complete eradication of prior HBV infection or elimination of the risk of HBV reactivation. Accordingly, anti-HBc seroclearance alone should not justify the modification of antiviral prophylaxis or virological monitoring strategies recommended for patients at risk of HBV reactivation [3,5,8].

Anti-HBc seroclearance has rarely been evaluated as a primary endpoint with serial anti-HBc measurements. Holtkamp et al. [9] reported persistent or intermittent anti-HBc loss in approximately 13% of patients with hematologic or oncologic diseases, 10% of solid organ transplant recipients, and 6% of people living with HIV, while Pasculli et al. [10] described intermittent anti-HBc loss in patients with multiple sclerosis receiving disease-modifying therapies. However, neither study specifically evaluated confirmed anti-HBc seroclearance in a predominantly rituximab-treated HBsAg-negative/anti-HBc-positive cohort. In our cohort, anti-HBc seroclearance occurred in 16.5% of patients, indicating that this phenomenon is not uncommon during anti-CD20 therapy.

The mechanisms underlying anti-HBc seroclearance have not yet been fully elucidated. Nevertheless, current evidence suggests that prolonged B-cell depletion induced by anti-CD20 therapy might play a central role in this process. Rituximab rapidly depletes CD20-positive B cells, including memory B cells, whereas long-lived plasma cells lacking CD20 expression are not directly targeted. As a result, circulating immunoglobulin concentrations are largely preserved during the early phase of treatment, whereas secondary humoral immune responses are substantially impaired [12,13,14].

Progressive declines in anti-HBc titers were observed both in patients who developed seroclearance and in those who remained anti-HBc-positive, suggesting a broad and sustained effect of anti-CD20 therapy on anti-HBc kinetics. This pattern suggests that anti-HBc seroclearance may represent the culmination of a gradual immunological process rather than an abrupt transition to seronegativity. As few studies have systematically characterized longitudinal anti-HBc kinetics during anti-CD20 therapy, our findings provide further insight into the long-term evolution of anti-HBc serology [9,10]. This is also clinically relevant in light of the findings of Yang et al. [15], who demonstrated that quantitative anti-HBc levels were associated with HBV reactivation risk in patients with lymphoma and resolved HBV infection.

Younger age was independently associated with anti-HBc seroclearance. Age-related alterations in humoral immunity, including changes in B-cell function, alterations in the memory B-cell compartment, and impaired humoral responses, may provide a biological explanation for this association [16,17]. However, data on the relationship between age and anti-HBc seroclearance remain scarce, and further studies are needed to clarify the underlying mechanisms.

In the present study, fewer cumulative anti-CD20 treatment cycles were independently associated with anti-HBc seroclearance. Although this finding may initially appear counterintuitive, differences in treatment strategies and clinical characteristics across disease populations may have contributed to this association. Thus, the observed relationship may reflect residual confounding related to differences in patient populations and treatment patterns rather than a direct effect of fewer treatment cycles on anti-HBc seroclearance.

Mycophenolate mofetil (MMF) use was also independently associated with anti-HBc seroclearance. MMF inhibits inosine monophosphate dehydrogenase, thereby suppressing guanosine nucleotide synthesis and selectively inhibiting the proliferation of T and B lymphocytes, which rely predominantly on the de novo purine synthesis pathway for proliferation [18,19]. In addition, MMF has been shown to suppress B-cell activation, plasma cell differentiation, and immunoglobulin production [19,20]. Given these biological effects, MMF may contribute to a more pronounced decline in anti-HBc antibody levels over time. Consistent with this hypothesis, our linear mixed-effects model demonstrated a significantly steeper decline in anti-HBc titers among patients receiving MMF. To our knowledge, however, no previous study has directly examined the association between MMF use and anti-HBc seroclearance. Accordingly, our findings should be considered hypothesis-generating and require confirmation via larger prospective studies with mechanistic investigations.

A history of hematopoietic stem cell transplantation (HSCT) has shown a borderline association with anti-HBc seroclearance [21,22]. Post-transplant immune reconstitution and remodeling of the B-cell compartment may influence HBV serological markers, as loss of HBV antibodies and HBsAg seroreversion have been reported following HSCT [23].

Baseline HBV DNA was available for the entire cohort. Although detectable HBV DNA was present in 10 patients (4.3%), none developed anti-HBc seroclearance, and the exclusion of these patients in a sensitivity analysis did not materially alter the observed seroclearance rate. One of the most clinically important findings of our study was that HBV reactivation occurred even in patients who developed anti-HBc seroclearance. Among the five patients who experienced HBV reactivation during follow-up, two exhibited confirmed anti-HBc seroclearance. One of these patients died of HBV-related acute-on-chronic liver failure following premature discontinuation of antiviral prophylaxis, whereas the other died from progression of the underlying hematologic malignancy. Although the small number of reactivation events precludes definitive conclusions regarding differences in reactivation risk according to anti-HBc seroclearance status, the occurrence of reactivation in two patients after documented seroclearance indicates that loss of anti-HBc should not be interpreted as evidence of elimination of reactivation risk [3,5,8].

This study has several important strengths. To our knowledge, it represents one of the largest cohorts to evaluate anti-HBc seroclearance as the primary endpoint in HBsAg-negative/anti-HBc-positive patients receiving anti-CD20 therapy. In addition, all anti-HBc seroclearance events were confirmed by subsequent serological assessment, and the availability of serial anti-HBc and anti-HBs measurements enabled a detailed characterization of the long-term kinetics of HBV serological markers. Finally, the simultaneous evaluation of serological changes, HBV reactivation, and clinically relevant outcomes substantially enhances the clinical relevance of our findings.

Several limitations should also be acknowledged. First, the retrospective, single-center design may limit the generalizability of our findings. Second, because HBV serological assessments were performed as part of routine clinical care rather than according to a standardized study protocol, serological measurements were not available for all patients at every follow-up time point. Information on HBV vaccination during follow-up was not systematically available; therefore, a potential effect of vaccination on longitudinal anti-HBs dynamics cannot be excluded. However, HBV vaccination would not be expected to affect anti-HBc seroclearance, which was the primary outcome of this study. In addition, IVIG administration may result in passive transfer of anti-HBc, potentially complicating the interpretation of subsequent seroclearance [11]. However, individual assessment of IVIG timing and a sensitivity analysis excluding patients with first documented anti-HBc positivity within 90 days after IVIG administration yielded essentially unchanged results, reducing the likelihood that passive antibody transfer materially influenced our findings. Death may also represent a competing event for anti-HBc seroclearance. Although 39 deaths were documented during the overall observation period, only three occurred while patients remained under observation and before documented seroclearance. Given the small number of such competing events, a formal competing-risk analysis was not performed, and this should be considered when interpreting the time-to-event estimates. Finally, the relatively small number of patients in certain subgroups, particularly those with a history of HSCT or those receiving MMF, warrants cautious interpretation of these subgroup analyses.

## 5. Conclusions

In conclusion, anti-HBc seroclearance is a more frequent serological phenomenon than previously recognized among HBsAg-negative/anti-HBc-positive patients receiving anti-CD20 therapy and is accompanied by a progressive decline in anti-HBc titers over time. Nevertheless, the occurrence of HBV reactivation in patients who develop anti-HBc seroclearance indicates that this serological change should not be interpreted as evidence of complete resolution of prior HBV infection. Accordingly, anti-HBc seroclearance alone does not support the modification of current antiviral prophylaxis or virological monitoring strategies. Larger prospective multicenter studies are warranted to validate these findings and to further elucidate the immunological mechanisms underlying anti-HBc seroclearance.

## Figures and Tables

**Table 1:** Baseline demographic, clinical, treatment-related, and laboratory characteristics of the study cohort.

Variable	Overall (n = 230)
Demographic characteristics	
Age, years	61 (52–67.8)
Male sex, n (%)	107 (46.5)
Follow-up duration, months	32.5 (18.5–57.5)
Underlying indication for anti-CD20 therapy, n (%)	
Hematologic disorders	82 (35.7)
Rheumatologic disorders	77 (33.5)
Nephrologic disorders	11 (4.8)
Neurologic disorders	47 (20.4)
Dermatologic disorders	16 (7.0)
Treatment-related characteristics	
Hematopoietic stem cell transplantation, n (%)	8 (3.5)
Intravenous immunoglobulin use, n (%)	38 (16.5)
Rituximab only, n (%)	214 (93.0)
Exposure to another anti-CD20 agent, n (%)	16 (7.0)
Number of anti-CD20 treatment cycles	6 (4–10)
Cytotoxic therapy, n (%)	108 (47.0)
Corticosteroid therapy, n (%)	192 (83.5)
Concomitant immunosuppressive therapy, n (%)	210 (91.3)
Mycophenolate mofetil therapy, n (%)	23 (10.0)
Antiviral prophylaxis, n (%)	230 (100.0)
Entecavir prophylaxis, n (%)	97 (42.2)
Tenofovir disoproxil fumarate prophylaxis, n (%)	39 (17.0)
Tenofovir alafenamide prophylaxis, n (%)	102 (44.3)
Lamivudine prophylaxis, n (%)	5 (2.2)
Baseline laboratory findings	
ALT (U/L)	19 (13–26.8)
AST (U/L)	19.5 (14.2–28)
Total bilirubin (mg/dL)	0.5 (0.4–0.75)
Albumin (g/L)	41 (37–44)
Creatinine (mg/dL)	0.76 (0.6–0.9)
White blood cell count (×10^9^/L)	7.92 (5.79–10.59)
Absolute neutrophil count (×10^9^/L)	4.93 (3.5–7.4)
Absolute lymphocyte count (×10^9^/L)	1.82 (1.15–2.65)
Hemoglobin (g/dL)	12.4 (11–13.6)
Platelet count (×10^9^/L)	233 (179–293.8)
Baseline anti-HBs positivity (≥10 mIU/mL), n (%)	114/221 (51.6)
Baseline anti-HBs titer (mIU/mL)	10.4 (4.2–213)
Baseline anti-HBc titer (COI)	5.8 (3–13.4)

Notes: Data are presented as median (interquartile range) or n (%), unless otherwise indicated. Follow-up duration was defined as the interval from anti-CD20 treatment initiation to the last available follow-up. Abbreviations: ALT, alanine aminotransferase; AST, aspartate aminotransferase; anti-HBs, antibody to hepatitis B surface antigen; anti-HBc, antibody to hepatitis B core antigen; COI, cutoff index; MMF, mycophenolate mofetil.

**Table 2:** Longitudinal dynamics of anti-HBc and anti-HBs titers according to anti-HBc seroclearance status.

Time Point	SC (+)			SC (−)		
	*n*	Anti-HBc, Median (IQR)	Anti-HBs, Median (IQR)	*n*	Anti-HBc, Median (IQR)	Anti-HBs, Median (IQR)
M0	38	4.0 [1.6–334.8]	8.1 [2.3–335.8]	192	6.0 [3.4–9.4]	10.4 [4.7–177.5]
Month 6	33	0.8 [0.1–1.6]	36.2 [5.7–289.0]	140	4.8 [3.3–6.4]	42.2 [6.1–389.2]
Month 12	21	0.3 [0.0–1.0]	29.6 [1.3–169.5]	119	4.9 [3.3–5.8]	40.5 [3.0–320.2]
Month 18	20	0.1 [0.1–0.7]	36.0 [4.1–201.5]	101	4.4 [2.6–6.3]	30.0 [3.0–230.0]
Month 24	14	0.2 [0.0–0.9]	69.1 [12.5–307.5]	73	4.4 [3.0–5.6]	28.4 [1.9–179.6]
Month 30	12	0.3 [0.1–0.6]	22.5 [3.4–101.9]	60	4.2 [2.6–5.7]	14.9 [1.5–110.6]
Month 36	12	0.1 [0.0–0.9]	8.4 [1.2–231.4]	58	4.0 [2.6–6.9]	16.1 [0.7–114.3]
Month 42	6	0.5 [0.2–0.7]	19.9 [3.5–228.4]	42	3.9 [2.5–6.4]	18.9 [1.5–118.0]
Month 48	7	0.0 [0.0–0.0]	37.7 [6.8–133.8]	30	4.7 [2.7–5.8]	9.6 [0.9–77.1]
Month 54	3	0.6 [0.3–3.6]	313.0 [160.6–656.5]	25	4.5 [2.8–6.6]	12.6 [0.8–150.8]
Month 60	2	3.4 [2.2–4.5]	148.1 [92.2–204.1]	25	4.0 [2.6–5.5]	5.8 [1.4–64.6]

Notes: Values are presented as median (interquartile range [IQR]). “*n*” represents the number of participants with available serological measurements at the corresponding time point. SC (+) denotes patients who developed confirmed anti-HBc seroclearance at any time during follow-up, whereas SC (−) denotes patients who remained persistently anti-HBc-positive throughout follow-up. Thus, the anti-HBc values shown in the SC (+) group represent longitudinal measurements from patients classified according to their eventual seroclearance status and do not indicate that all patients were anti-HBc-negative at each individual time point. Measurements beyond month 24 are presented as descriptive exploratory data because of the progressively decreasing number of available observations and were not included in the primary longitudinal mixed-effects analyses.

**Table 3:** Univariable and multivariable Cox proportional hazards regression analysis of factors associated with anti-HBc seroclearance.

Predictor	Univariable		Multivariable	
	HR (95% CI)	*p*	HR (95% CI)	*p*
Age (years)	0.95 (0.92–0.97)	<0.001	0.96 (0.93–0.98)	0.001
Male sex	1.38 (0.71–2.67)	0.342	–	–
Total anti-CD20 treatment cycles	0.88 (0.81–0.96)	0.004	0.90 (0.83–0.99)	0.025
HSCT history	2.85 (1.01–8.03)	0.048	2.89 (0.97–8.64)	0.057
Hematologic disease presence	1.24 (0.64–2.41)	0.522	–	–
Rheumatologic disease category (overall variable)				
Rheumatoid arthritis vs. no rheumatologic disease	0.23 (0.07–0.76)	0.016	–	–
ANCA-associated vasculitis vs. no rheumatologic disease	0.47 (0.11–1.95)	0.296	–	–
Other autoimmune rheumatologic disease vs. no rheumatologic disease	0.33 (0.05–2.42)	0.276	–	–
Neurologic disease category (overall variable)				
Multiple sclerosis vs. no neurologic disease	0.57 (0.20–1.62)	0.292	–	–
Other neurologic diseases vs. no neurologic disease	3.64 (1.27–10.42)	0.016	–	–
Cytotoxic therapy	0.92 (0.48–1.75)	0.792	–	–
Corticosteroid therapy	0.76 (0.35–1.67)	0.497	–	–
Mycophenolate mofetil therapy	3.44 (1.62–7.29)	0.001	2.52 (1.14–5.56)	0.022
IVIG therapy	3.04 (1.55–5.95)	0.001	–	–
Anthracycline chemotherapy	0.96 (0.45–2.04)	0.918	–	–
Entecavir	0.70 (0.36–1.36)	0.291		
Tenofovir disoproxil fumarate	0.34 (0.10–1.10)	0.070		
Tenofovir alafenamide	2.03 (1.06–3.89)	0.033		
Lamivudine	Not estimable *	–		
Baseline HBV-DNA status	Not estimable **	–	–	–
Baseline lymphocyte count	1.00 (0.99–1.01)	0.690	–	–
Baseline ALT	1.00 (0.98–1.02)	0.823	–	–
Baseline creatinine	1.22 (0.93–1.59)	0.144	–	–
Log10 (baseline anti-HBc titer + 1)	1.33 (0.93–1.90)	0.118	–	–
Log10 (baseline anti-HBs titer + 1)	0.96 (0.66–1.38)	0.814	–	–

* Hazard ratios for lamivudine exposure could not be reliably estimated because no anti-HBc seroclearance events occurred among the five patients exposed to lamivudine. ** The hazard ratio for baseline HBV-DNA positivity could not be reliably estimated because no anti-HBc seroclearance events occurred among the 10 patients with detectable baseline HBV DNA. Abbreviations: HR, hazard ratio; CI, confidence interval; HSCT, hematopoietic stem cell transplantation; IVIG, intravenous immunoglobulin.

**Table 4:** Longitudinal changes in anti-HBc titers during the first 24 months after anti-CD20 therapy initiation.

Follow-Up Time	*n*	Anti-HBc Titer, Median (IQR)	
Baseline	227	5.70 (2.90–12.90)	
Month 6	170	4.30 (2.30–6.14)	
Month 12	137	4.30 (1.90–5.70)	
Month 18	120	3.99 (1.77–5.82)	
Month 24	86	3.85 (2.02–5.38)	
**Linear Mixed-Effects Model Results**			
**Effect**	**Estimate**	**F Value**	***p* Value**
Follow-up time	−0.0157	88.36	<0.001
MMF exposure	0.4331	21.24	<0.001
Follow-up time × MMF exposure	−0.0292	20.58	<0.001

Note. Anti-HBc titers are presented as median (interquartile range). Longitudinal changes were evaluated using a linear mixed-effects model with patient-specific random intercepts.

**Table 5:** Longitudinal changes in anti-HBs titers during the first 24 months after anti-CD20 therapy initiation.

Follow-Up Time	*n*	Anti-HBs Titer, Median (IQR)	
Baseline	226	10.1 (4.12–192.0)	
Month 6	171	38.3 (5.85–361.0)	
Month 12	134	39.1 (2.35–300.0)	
Month 18	121	30.0 (3.0–230.0)	
Month 24	87	30.0 (2.36–240.0)	
**Linear Mixed-Effects Model Results**			
**Effect**			
Follow-up time		5.41	0.020
MMF exposure		1.17	0.280
Follow-up time × MMF exposure		6.74	0.010

Note. Anti-HBs titers are presented as median (interquartile range). Longitudinal changes were evaluated using a linear mixed-effects model with patient-specific random intercepts.

**Table 6:** Characteristics and clinical outcomes of patients with HBV reactivation.

Patient	Underlying Disease (Age/Sex)	Antiviral Agent Used for Prophylaxis	Prophylaxis Ongoing at Reactivation	Time to Reactivation (Months)	HBV DNA at Reactivation (IU/mL)	HbsAg Seroreversion	Peak ALT (U/L)	HBV-Related Hepatitis	Anti-HBc Status	Management	Outcome
1	Polyarteritis nodosa 63/M	Lamivudine	Yes	52	24,120,353	Yes	1021	Yes	9.22 S/CO	Switched to TAF; achieved HBsAg loss.	Alive; off antiviral therapy after HBsAg loss
2	Multiple sclerosis 69/M	TAF	Yes	18	Negative	Yes	47	No	5.41 S/CO	Continued antiviral prophylaxis.	Alive; no HBV-related hepatitis during 16 months of follow-up
3	Burkitt lymphoma 52/M	Entecavir	No *	17	709,628,151	Yes	923	Yes	17.56 S/CO	Antiviral prophylaxis was interrupted; HBV-related hepatitis developed.	Liver-related death (HBV-related hepatitis)
4	Mantle cell lymphoma 65/M	TAF	No	19	39,664,051	Yes	750	Yes	0.05 S/CO (seroclearance)	Antiviral prophylaxis was discontinued before completion of rituximab therapy.	Liver-related death (ACLF)
5	Mantle cell lymphoma 71/M	TAF	No *	32	11	Yes	49	No	0.75 S/CO (seroclearance)	No additional antiviral intervention.	Death due to underlying hematologic malignancy

* Antiviral prophylaxis was interrupted or discontinued before completion of anti-CD20 therapy. Abbreviations: ACLF, acute-on-chronic liver failure; ALT, alanine aminotransferase; HBsAg, hepatitis B surface antigen; HBV, hepatitis B virus; S/CO, signal-to-cutoff ratio; TAF, tenofovir alafenamide. Liver-related death was defined as death attributable to HBV-related hepatitis or acute-on-chronic liver failure (ACLF).

## Data Availability

The data presented in this study are available from the corresponding author upon reasonable request. The data are not publicly available due to privacy and ethical restrictions related to patient confidentiality.

## Abbreviations

The following abbreviations are used in this manuscript:

ACLF	Acute-on-Chronic Liver Failure
ALT	Alanine Aminotransferase
ANCA	Antineutrophil Cytoplasmic Antibody
anti-HBc	Antibody to Hepatitis B Core Antigen
anti-HBs	Antibody to Hepatitis B Surface Antigen
AST	Aspartate Aminotransferase
CI	Confidence Interval
CLL	Chronic Lymphocytic Leukemia
ETV	Entecavir
HBsAg	Hepatitis B Surface Antigen
HBV	Hepatitis B Virus
HR	Hazard Ratio
HSCT	Hematopoietic Stem Cell Transplantation
IQR	Interquartile Range
IVIG	Intravenous Immunoglobulin
MMF	Mycophenolate Mofetil
NHL	Non-Hodgkin Lymphoma
RA	Rheumatoid Arthritis
RTX	Rituximab
S/CO	Signal-to-Cutoff Ratio
TAF	Tenofovir Alafenamide
ULN	Upper Limit of Normal
